# Molecular stratification within triple-negative breast cancer subtypes

**DOI:** 10.1038/s41598-019-55710-w

**Published:** 2019-12-13

**Authors:** Dong-Yu Wang, Zhe Jiang, Yaacov Ben-David, James R. Woodgett, Eldad Zacksenhaus

**Affiliations:** 1 Toronto General Research Institute, University Health Network, 67 College Street, Toronto, Ontario M5G 2M1 Canada; 2 The Key laboratory of Chemistry for Natural Products of Guizhou Province and Chinese Academic of Sciences, Guiyang, Guizhou 550014 China; 3 State Key Laboratory for Functions and Applications of Medicinal Plants, Guizhou Medical University, Guiyang, 550025 China; 4 Lunenfeld-Tanenbaum Research Institute, Sinai Health System, 600 University Avenue, Toronto, ON Canada; 5 Department of Medicine, University of Toronto, Toronto, Ontario Canada

**Keywords:** Breast cancer, Genome informatics

## Abstract

Triple-negative breast cancer (TNBC) has been subdivided into six distinct subgroups: basal-like 1 (BL1), basal-like 2 (BL2), mesenchymal (M), mesenchymal stem–like (MSL), immunomodulatory (IM), and luminal androgen receptor (LAR). We recently identified a subgroup of TNBC with loss of the tumor suppressor PTEN and five specific microRNAs that exhibits exceedingly poor clinical outcome and contains TP53 mutation, RB1 loss and high MYC and WNT signalling. Here, show that these PTEN-low/miRNA-low lesions cluster with BL1 TNBC. These tumors exhibited high RhoA signalling and were significantly stratified on the basis of PTEN-low/RhoA-signalling-high with hazard ratios (HRs) of 8.2 (P = 0.0009) and 4.87 (P = 0.033) in training and test cohorts, respectively. For BL2 TNBC, we identified AKT1 copy gain/high mRNA expression as surrogate for poor prognosis (HR = 3.9; P = 0.02 and HR = 6.1; P = 0.0032). In IM, programmed cell death 1 (PD1) was elevated and predictive of poor prognosis (HR = 5.3; P = 0.01 and HR = 3.5; P < 0.004). Additional alterations, albeit without prognostic power, characterized each subtype including high E2F2 and TGFβ signalling and CXCL8 expression in BL2, high IFNα and IFNγ signalling and CTLA4 expression in IM, and high EGFR signalling in MSL, and may be targeted for therapy. This study identified PTEN-low/RhoA-signalling-high, and high AKT1 and PD1 expression as potent prognostications for BL1, BL2 and IM subtypes with survival differences of over 14, 2.75 and 10.5 years, respectively. This intrinsic heterogeneity could be exploited to prioritize patients for precision medicine.

## Introduction

Breast Cancer (BC) is pathologically classified as oestrogen-positive (ER^+^), HER2/ERBB2/NEU-positive (HER2^+^) and triple negative (TNBC) subtypes^1–3^. The latter group represents ~15% of all BC cases but has poor prognosis and affects young women with a dramatically higher incidence in African and African-American women^4,5^. TNBC can be sub-divided into 6 subtypes: basal-like (BL1 and BL2), mesenchymal (M), mesenchymal stem-like (MSL), immunomodulatory (IM), and luminal androgen receptor (LAR), as well as an unspecified group (UNS)^3^. An alternative classification divides TNBC into BL1 and BL2, M and LAR^6^. Whether these subgroups can be further stratified, for example through multi-omic approaches^7^, is largely unknown.

Approximately 20% of TNBC patients respond well to standard therapy (tumor resection, radiation and cytotoxic chemotherapy), but the rest develop lethal metastatic disease. A recent clinical trial has demonstrated an encouraging response of some TNBC patients to the immune-checkpoint blockade (ICB) inhibitor, Atezolizumab (anti-PD-L1), in combination with conventional chemotherapy^8^ (Reviewed in^9^). Yet, most patients, including those with high expression of PD-L1, succumbed to the disease. The identification of TNBC subtype that are likely to respond to ICB therapy is of great interest.

Germ line mutations in the tumor suppressor genes BRCA1 and BRCA2 lead to basal-like BC^10–12^; and many TNBC with intact BRCA1/2 are nonetheless classified as BRCAness lesions^13^. PARP inhibitors are synthetic lethal with BRCA1/2 mutant/BRCAness TNBC and have been approved for therapy^14^. However, emergence of clones that resist PARP inhibition through multiple mechanisms is a major clinical problem^15^. Genomic analysis of sporadic TNBC identified combined loss of RB1 (mutation/deletion; promoter/mRNA silencing) plus TP53 (mutation/deletion) in as many as 28–40% of cases^16–19^. Additional alterations include PTEN loss or PIK3CA mutation, and enhanced EGFR, WNT and MYC signaling. RB1, PTEN and TP53 are also the most frequent drivers of metastasis in diverse types of solid human cancers including breast cancer^20^.

We recently demonstrated that inactivation of Pten in the mouse mammary gland induces mammary tumors that fail to induce secondary tumors after orthotopic injection into recipient mice^21^. An exception was a relatively rare group of tumors resembling basal-like BC, which could be efficiently propagated in recipient mice. These transplantable mouse tumors exhibited low expression of the tumor suppressor microRNA-145, raising the question of whether in breast cancer patients, PTEN-deficiency cooperates with miR-145 loss and/or other microRNAs to define an aggressive subgroup of TNBCs. We found that low expression of four of the following microRNAs: hsa-miR-145, hsa-miR-4324, hsa-miR-125b, hsa-miR-381 and hsa-miR136 in cooperation with PTEN loss marks highly aggressive TNBCs. These PTEN-low/miRs-low TNBCs exhibit TP53 mutation (not deletion), loss of RB1 signature, and high MYC, WNT and PI3K signalling.

Here we sought to determine whether these PTEN-low/miRs-low TNBCs are spread among different TNBC subtypes or concentrate within one particular subgroup. We found that these tumors cluster almost exclusively with the BL1 subtype and represent lesions with the poorest clinical outcome. Further analysis revealed that this aggressive subgroup of BL1 TNBC exhibit unique signalling attributes such as high RhoA pathway activity. Furthermore, PTEN-low/Rho-signalling high BL1 TNBC patients had the worst prognosis. This observation indicates that the broad survival curve of BL1 patients (and by extension, other subtypes) is not merely stochastic in nature but a reflection of intrinsic heterogeneity that can be molecularly identified and potentially exploited therapeutically. These findings prompted further analysis of other TNBC subtypes and the identification of AKT1 copy number gain and high expression as predictor of poor prognosis in BL2, and high Programmed Cell Death 1 (PD1) expression as predictor of poor prognosis in IM TNBC. This and additional analysis presented herein provide evidence that patients within each TNBC subtype can be molecularly stratified and prioritized for targeted therapy.

## Results

### Pten-low/miR-low TNBCs cluster with BL1 lesions with exceedingly poor clinical outcome

**Figure 1:**
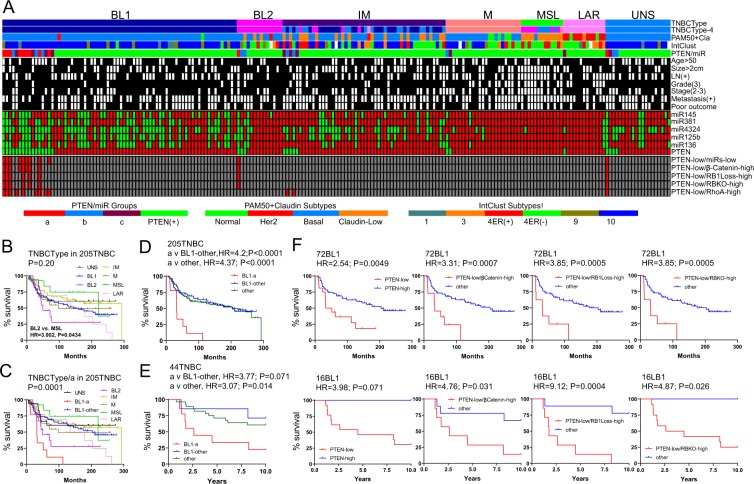
Most PTEN-low/miR-low (group-a) tumors cluster with the BL1 TNBC subtype. (**A**) Classification of 205 TNBCs into six Lehmann TNBC subtypes: basal-like 1 (BL1), basal-like 2 (BL2), mesenchymal (M), mesenchymal stem–like (MSL), immunomodulatory (IM), and luminal androgen receptor (LAR) as well as unspecified tumors (UNS). Also shown are the four TNBC subtypes; PAM50 + Claudin-low; IntClust, PTEN-low/miR-low and the BL1-group “a” subgroup. Pathological data, miRNA expression and classification of PTEN-low/βCatenin-high, PTEN-low/RB1Loss-high, PTEN-low/RBKO-high, and PTEN-low/RhoA-high are indicated. (**B**,**C**) Survival of six TNBC subtypes as well as unspecified TNBC tumors (UNS) in a cohort of 205 TNBCs. In C, BL1 is stratified into BL1-group-“a” versus all other BL1 lesions. (**D**) Survival of BL1-group-“a” versus all other BL1 lesions or all other TNBC subtypes and corresponding hazard ratios (HRs) in the 205 TNBC cohort. **(E)** Survival of BL1-group-“a” versus all other BL1 lesions or all other TNBC subtypes and corresponding HRs in a cohort of 44 TNBCs. **(F**) Survival of 72 and 16 BL1 TNBCs in both the training **(D)** and validation **(E)** sets. Low PTEN expression in BL1 correlated with poor prognosis in both 72 BL1 tumors from the training set and 16 BL1 tumors from the validation set. Low PTEN expression and high pathway activity of β-Catenin, or loss of RB1 using two different signatures RBKO (RB sig. loss and RB knockout) predicted poor clinical outcome in both cohorts.

We first classified a publically available dataset of 205 TNBCs with mRNA and miRNA expression and clinical data into 6 (BL1, BL2, M, MSL, IM, LAR) or 4 (BL1 and BL2, M and LAR) subtypes as described by Lehmann *et al*.^3,6^, (Fig. 1A). TNBCs that did not cluster with any of these subtypes were designated UNS (unspecified). In addition, “PAM50 + cla” classification identified most TNBCs as Basal-like or Claudin-low (cla) lesions, as well as some Normal-like and HER2+ tumors. We then asked how PTEN-low and PTEN-low/miRNA-low (group “a”) TNBCs clustered with respect to these TNBC subtypes. Nine of the eleven PTEN-low/miRNA-low TNBCs grouped together with BL1. The remaining two tumors clustered with BL2 and UNS, though the one that classified with UNS (r = 0.5454; p < 0.0001) by correlation to TNBCType centroids was very close to BL1 (r = 0.5188; p < 0.0001).

Genomic and transcriptomic analysis has stratified breast cancer into 10 intrinsic subtypes with IntClust10 comprising most basal-like breast cancers^16,22^. Consistent with this, all PTEN-low/miRNA-low TNBCs that were classified as BL1 also clustered with IntClust10 (Fig. 1A). Notably, the UNS group showed similar PAM50 and IntClust10 clustering as BL1. In addition, while BL-1 group “a” had poor outcome, other pathological parameters were similar across the different TNBC subtypes (black boxes in Fig. 1A).

Kaplan-Meier survival curves for the six Lehmann TNBC-subtypes as well as UNS tumors are shown in Fig. 1B. BL1 TNBCs in this dataset are second most aggressive after BL2. When BL1 tumors were divided according to PTEN and miRNA status, the PTEN-low/miRNA-low BL1 subgroup (group “a”) exhibited the worst clinical outcome compared to all other BL1 or other TNBC subtypes including BL2, with hazard ratios (HRs) of 4.2 and 4.37, respectively (P < 0.0001; Fig. 1C,D). Using a validation cohort of 44 TNBC, PTEN-low/miRNA-low TNBCs also clustered with BL1 and stratified this subgroup into poor and relatively good clinical outcome; HR for the PTEN-low/miRNA-low subgroup compared with the other BL1 lesions, or relative to all other TNBCs was 3.77 (P = 0.071) and 3.07 (P = 0.014), respectively (Fig. 1E). Specifically, the median latency was 32 and 31.4 months for PTEN-low/miRNA-low BL1 patients versus 204 months or undefined period (i.e. % survival not crossing the 50% mark) for all other BL1 patients in the two cohorts.

We previously showed that PTEN-low/miRNA-low TNBCs exhibited high WNT signalling and loss of RB1 signature^23^. We therefore asked whether these two classifiers were also able to stratify BL1 patients. Using two different signatures for RB1 loss^19,23–25^, PTEN-low/RB-loss and PTEN-low/β-catenin-high TNBCs almost completely overlapped with the PTEN-low/miRNA-low subgroup (Fig. 1A). Moreover, these classifiers significantly stratified BL1 patients into poor and good clinical outcomes on both test and validation cohorts with high hazard ratios of 3.3 and 4.7 for PTEN-low/β-catenin pathway high, and 3.8 to 9.1 for PTEN-low/RB-signature-low (Fig. 1F). Thus, in two independent cohorts, PTEN-low/miRNA-low TNBCs cluster with BL1 and represent patients with extremely poor prognosis. Moreover, these patients can be identified using PTEN-low and either β-catenin high or RB1-loss signatures.

### Unique signalling pathways in different TNBC subtypes; high RhoA signalling predicts poor clinical outcome in BL1 patients

**Figure 2:**
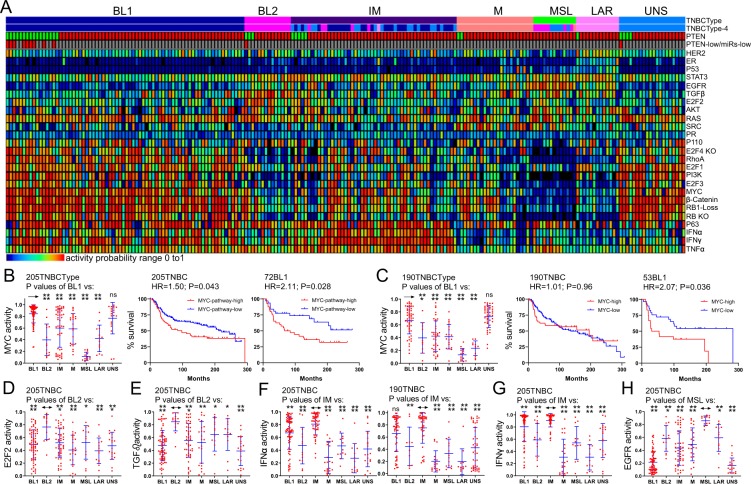
Identification of signaling pathways that are altered in each specific TNBC subtypes. (**A**) Classification of 205 TNBCs into subtypes, and activity of 25 different signaling pathways. (**B**,**C**) In both training and validation sets high MYC pathway activity alone stratifies BL1 TNBC into high and low risk groups. **(D**,**E)** E2F2 and TGFβ pathways are significantly elevated in BL2. (**F**,**G**) IFNα and IFNγ pathways are significantly induced in IM. **(H)** EGFR activity is significantly higher in MLS compared to all other TNBC subtypes. Only IFNα signalling affected clinical outcome (Supplemental Fig. 1).

The aforementioned results showing that BL1 TNBC patients can be molecularly stratified into good-poor prognosis groups raised the question of whether other TNBC subtypes might also be clinically stratified. To identify Lehmann TNBC subtype-specific alterations, we first determined activity for twenty-five pathways/signatures using established conditions^25,26^ (Fig. 2A). Consistent with our previous study, BL1 and particularly PTEN-low/miRNA-low BL1 lesions exhibited RB1-loss signature and high WNT and MYC signalling. Within BL1, the MYC signature alone sufficed to stratify this subtype into poor and moderate clinical outcome with HRs of ~2.1 in the test (205 patient) and validation (190 patient) cohorts (P = 0.027; P = 0.036; Fig. 2B,C). The validation cohort (190 patients) included 146 TNBCs from METABRIC (excluding the 205 TNBC samples in the test cohort) and the 44 TNBCs in cohort GSE22220 (both Illumina platforms).

Several signalling pathways were elevated in specific TNBC subtypes (Fig. 2D–H). Thus, E2F2 and TGFβ pathways were specifically elevated in BL2; IFNα and IFNγ pathways were high in IM; and EGFR signaling was prominent in MLS relative to other subtypes. With the exception of IFNα in one clinical cohort, none of these pathways had any prognostication value in the respective TNBC subtype (Supplemental Fig. 1). Nonetheless, these pathways represent specific markers and potential targets for therapy (see Discussion).

**Figure 3:**
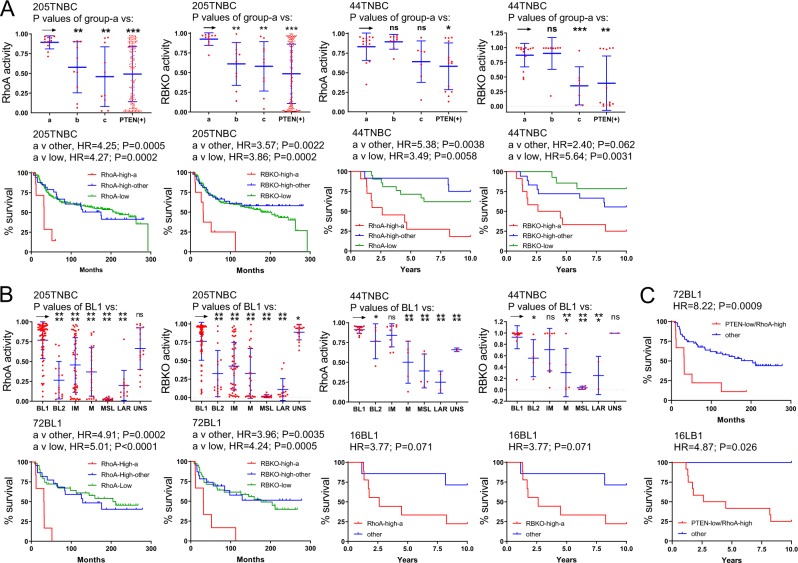
Expression levels and prognostic power of RhoA signaling and RB loss in TNBC subtypes. (**A)** High pathway activities of RhoA and RBKO pathways in PTEN-low/miR-low (group-“a”) vs PTEN-low/miRNA high (group “c”), vs all other PTEN-low TNBC (group “b”). PTEN(+) includes all PTEN-high TNBC samples. RhoA and RBKO pathways are elevated in group-a and exhibit poor clinical outcome in both the training and validation sets. (**B)** High pathway activities of RhoA and RBKO pathways in TNBC subtypes. Both pathways are elevated in BL1 and predict poor clinical outcome in the training and validation sets. (**C**) Exceedingly poor survival of BL1 TNBCs with low PTEN expression and high RhoA pathway activity.

Importantly, RhoA signalling was significantly elevated in PTEN-low/miRNA-low (group “a”) TNBC, compared with PTEN-low/miRNA-high (group “b”), PTEN-low/miRNA-others (group “c”) or PTEN-high (Fig. 3A), or in BL1 (Fig. 3B) vs most other subtypes in two different cohorts. Moreover, high RhoA signalling identified TNBCs with exceedingly poor clinical outcome (Fig. 3A,B). High RhoA signalling was as efficient as or an even better predictor than RB1-loss in identifying TNBC with poor prognosis. Moreover, in combination with PTEN status, PTEN-low/RhoA-high BL1 TNBC showed the poorest clinical outcome compared with all other BL1 lesions with HRs of 8.2 (P = 0.0009) and 4.87 (P = 0.025) in the two clinical cohorts (Fig. 3C). Median latency was 32 and 42.7 months for PTEN-low/RhoA-high BL1 relative to 204 or undefined period for all other BL1 patients in the two cohorts – a difference of over 14 years. Thus, RhoA signalling represents another targetable pathway that is elevated in BL1 and, together with PTEN-low status, predictive of poor outcome.

### Unique spectrum of mutations and gains/losses in different TNBC subtypes

To ask whether other TNBC subtypes can be molecularly stratified into tumors with distinct clinical outcomes, we analyzed the spectrum of mutations and copy number alterations (CNAs) in each subtype. We identified 135 genes with at least one mutation in 185 TNBCs (Supplemental Fig. 2). Only TP53 and PIK3CA were frequently mutated (Supplemental Fig. 3A,B). TNBC samples showing no TP53 mutation likely harbor p53 deletion or amplification of HDM2, the E3 ligase for this tumor suppressor^27^. Indeed, pathway activity for p53 showed loss of this tumor suppressor signaling in nearly all TNBCs, including all BL1, BL2 and IM (Fig. 2A). In accordance, TP53 mutation alone did not predict poor prognosis (not shown). In contrast, TNBCs with PIK3CA mutation showed significantly worst prognosis relative to TNBCs with wild-type PIK3CA in two different cohorts (Supplemental Fig. 3C). Furthermore, TNBCs selected on the basis of combined PIK3CA plus TP53 mutations showed similar poor prognosis as those selected on the basis of PIK3CA mutation alone (Supplemental Fig. 3D).

CNA analysis identified alterations using 22544 probes in all TNBC subtypes with the exception of MSLs (arrow in Supplemental Fig. 4). In search for specific regions that are gained or lost in each TNBC subtype using ANOVA-test-selected 2744 CNA probes (Supplemental Fig. 5), we identified common gains in chromosome 16p13.3 in BL2. This region contains 169 genes, of which, 12 are shown in Supplemental Fig. 6. These include 3-Phosphoinositide Dependent Protein Kinase 1 (PDPK1 also known as PDK1), which phosphorylates and activates a subgroup of AGC protein kinases like protein kinase B (PKBα/AKT1, PKBβ/AKT2, PKBγ/AKT3) downstream of PI3K. Chr16p13.3 gain is implicated in prostate cancer progression via induction of PDPK1^28^. However, although PDPK1 mRNA levels are significantly higher in BL2 compared to other subtypes, its expression did not correlate with poor prognosis in all TNBCs or specifically within BL2 (Supplemental Fig. 6). Likewise, expression of other genes and microRNAs such as miR3176, located within the 16p13.3 amplicon, was also elevated in BL2, but did not predict clinical outcome.

### High copy number alteration (CNA) and mRNA expression of AKT1 predict poor clinical outcome in BL2 TNBC subtype

**Figure 4:**
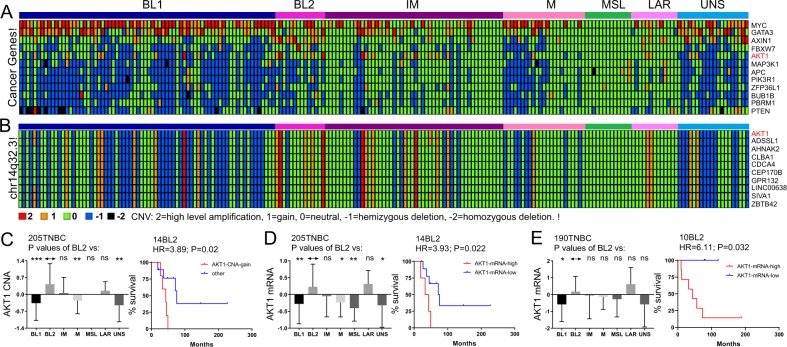
Copy number alterations (CNAs) in TNBC subtypes and identification of AKT1 gain and high expression as prognostic markers in BL2. (**A**) CNA in breast cancer related oncogenes and tumor suppressors. Note CNAs in AKT1 in several BL2 TNBC samples. (**B**) CNA in AKT1 and surrounding genes on chromosome 14q32.3. (**C**) AKT1 showed the most frequent high CNA gain that predicts poor outcome in BL2 subtype. (**D**,**E**) High mRNA expression of AKT1 predicts poor prognosis in BL2 tumors from both training and validation cohorts.

Compilation of CNA, exome and whole genome sequencing analysis identified 94 common oncogenic alterations in breast cancer^18^. Having found no obvious large CNA associated with poor prognosis, we asked whether any of the 94 common oncogenic alterations in breast cancer are associated with CNA in specific TNBC subtypes. Twelve such genes were identified including MYC, GATA3, AXIN1, FBXW7, AKT1, MAP3K1, APC, PIK3R1, BULB1B, PBRM1 and PTEN (Fig. 4A) from the ANOVA-test-selected CNA probes. Of these, only AKT1 copy number gain was associated with poor clinical outcome in BL2 with HR = 3.89 (P = 0.02; Fig. 4C; Supplemental Fig. 7). AKT1 is located on chromosome 14q32.3, a region of gain in multiple BL2 TNBC lesions (Fig. 4B). CNAs in neighbouring genes such as CDCA4, GPR132 and SIVA also predicted poor clinical outcome in BL2 (Supplemental Fig. 8A–C).

We next determined the effect of AKT1 mRNA expression on clinical outcome in BL2 TNBC. Using the 205 TNBC cohort with 14 BL2 tumors, high AKT1 expression identified patients with extremely poor prognosis compared with AKT1-low BL2 samples with HR = 3.93 (P = 0.022; Fig. 4D). The validation cohort with 190 TNBCs contained 10 BL2 samples that could be segregated on the basis of AKT1 expression with HR = 6.1 (P = 0.032; Fig. 4E). The median latency in the 14 BL2 and 10 BL2 cohorts was 38 and 43.4 months for high AKT1 expression versus 73.65 months and undefined period for low AKT1 expression. Thus, AKT1 copy number gain and high mRNA expression can be used to detect a subgroup of BL2 TNBCs with exceedingly poor prognosis. These tumors are likely sensitive to AKT1 inhibitors.

CDCA4 showed a similar profile as AKT1 in BL2 with significant HR for CNA, but its mRNA level stratified BL2 patients only in one of two cohorts (Supplemental Fig. 8A). Notably, expression of AKT1 and CDCA4 was highest in LAR and BL1, respectively, but they showed significant prognostic power only in BL2, in which copy number gain occurs. Several other genes in the chr14q32.3 amplicon in BL2 such as CLBA1 also predicted poor clinical outcome in the test cohort but only showed a trend toward significance in the validation cohort (Supplemental Fig. 8B,C). Thus, high copy number and mRNA expression of AKT1 are the only alterations that consistently showed significant prognostic power in all BL2 cohorts (CNA, mRNA).

### Expression analysis of immune-checkpoint blockade and inflammation genes identifies PD1 as prognostic marker for IM TNBC

As noted, some TNBC patients respond well to the anti-PD-L1 immune-checkpoint blockade (ICB) inhibitor, Atezolizumab, in combination with conventional chemotherapy^8^, yet, most patients, including those with PD-L1^+^ tumors, succumb to the disease. Here we asked whether ICB proteins or other inflammatory chemokines and cytokines are elevated in specific TNBC subtypes. We first examined their expression in PTEN-low/miRNA-low (group “a”) BL1 TNBCs versus all other TNBC, ER^+^ and HER2^+^ breast cancer samples (Supplemental Fig. 9A). The MYC pathway, PD-L1 (CD274) and CD47 were elevated in TNBC, particularly in PTEN-low/miRNA-low TNBCs (group “a”), relative to HER2^+^ or ER^+^ breast cancer in most cohorts (Supplemental Fig. 9B–D). Notably, MYC transcriptionally regulates expression of PD-L1 (CD274) and CD47^29^. CCL7 and CSF2 exhibited a significant increase in expression in group “a” vs TNBC, ER^+^ and HER2^+^ breast cancer samples, whereas CXCL8, ICAM1, CCL8, IGFBP3, CXCL1, CCL13, CCL2 and IL6 were elevated in group “a” relative to ER^+^ and HER2^+^ lesions (Supplemental Fig. 9E).

**Figure 5:**
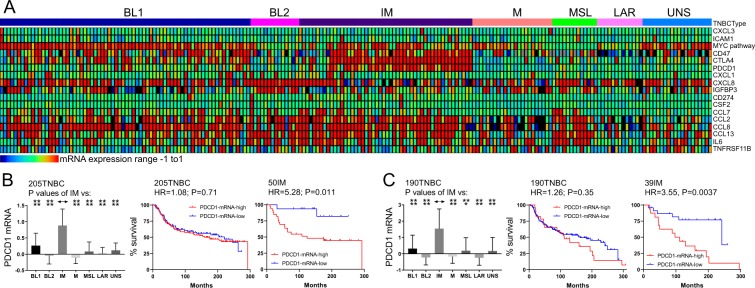
Stratification of IM TNBCs by expression of the immune checkpoint blockade gene PDCD1 (PD1). (**A)** Expression of immune checkpoint and inflammation associated genes in TNBC subtypes. (**B**,**C**) PDCD1 expression is elevated in IM TNBC subtype and predicts poor prognosis both in the training (205) and validation (190) TNBC cohorts.

Within TNBC subtypes, multiple ICB and inflammatory genes exhibited subtype specificity (Fig. 5A). The ICB genes PDCD1 (PD1) and CTLA4 were highly expressed in most IM TNBC samples. Importantly, PDCD1 expression predicted poor clinical outcome in two independent cohorts with HRs of 5.27 (P = 0.011) (Fig. 5B) and 3.55 (P = 0.0037) (Fig. 5C). Median latency for high PDCD1 IM patients in these cohorts was 124 and 104 months compared with undefined period or 241.5 months for IM patients expressing low level of PDCD1, respectively – a difference of over 10 years. In contrast, PD1 expression had little prognostic power in all TNBC lesions indicating that other factors in the immune microenvironment likely impact outcome in the other subtypes (see Discussion). High CTLA4 expression showed HR of 4.19 but the P value only showed a trend towards significance (P = 0.072) possibly due to a small number of patients in the CTLA4-negative arm (Supplemental Fig. 10A). In addition, CCL2 was highly expressed in most MSL, whereas, CXCL8 was elevated in all BL2 but with no prognostic value (Supplemental Fig. 10B,C).

## Discussion

The development of effective therapy for TNBC is hampered in part by the high level of heterogeneity in these tumors. Indeed, TNBC patients are pathologically defined in negative terms by their low expression of ER, PR and HER2, not positively via specific markers. BRCA1/2 mutations and BrCAness are a hallmark of familial basal-like breast cancer, but sporadic TNBCs are poorly defined in the clinic. This low information state has been improved by molecular classifications that initially defined basal and claudin-low TNBC and more recently six different TNBC subtypes: basal-like 1 (BL1), basal-like 2 (BL2), mesenchymal (M), mesenchymal stem–like (MSL), immunomodulatory (IM), and luminal androgen receptor (LAR)^3^. Each of these TNBC subgroups shows a wide range of survival over 20 years from initial detection, suggesting either that (i) actual death may be stochastic or affected by extrinsic factors such as overall health status and treatment regimens (e.g. successful/timely removal of primary tumor prior to dissemination), or (ii) each subtype is inherently heterogeneous and driven by different oncogenic alterations within each subtype. Here we provide evidence for the latter scenario; we show that BL1, BL2 and IM TNBC can be stratified on the basis of specific oncogenic alterations into poor and relatively moderate subgroups. High risk BL1 tumors show multiple alterations including high WNT, MYC, and RhoA signalling and loss of RB1 and PTEN plus p53 mutation rather than deletion; high risk BL2 lesions exhibit AKT1 copy number gain and high AKT1 mRNA expression, whereas high risk IM patients show high mRNA expression of the ICB gene PDCD1 (PD1).

The difference in survival between TNBCs with these alterations compared with those without them is remarkable. For BL1, the average median difference is <32 vs >204 months for group “a” or ~36 vs >204 for Pten-low/RhoA signaling high (a greater than 14 year difference); for BL2 it is <41 vs >74 months for AKT1 high (>2.75 year difference); and for IM, 114 vs >242 months for high PD1 (>10.67 year difference). Thus, the wide range of survival in each subtype of TNBC patients is driven by intrinsic heterogeneity and specific oncogenic alterations. Upon further validation of these findings, these patients can be identified and prioritized for therapy.

The oncogenic alterations that stratify BL1, BL2 and IM offer potential targets for therapeutic intervention. Thus, high risk BL1 may be treated with inhibitors of the PI3K pathway, WNT, MYC, RhoA and/or RB1 downstream effectors^30^; BL2 with AKT1 inhibitors, and IM with PD1/PD-L1 based therapy. Further analysis may identify oncogenic alterations that can stratify M, MSL and LAR TNBC subtypes. We also found specific alterations in each subtype that did not stratify these patients into different risk groups, but may serve as excellent targets for therapy. Moreover, these genes are candidates for the creation of new genetically engineered mouse models for testing TNBC stratification and aggressiveness, as well as novel therapies.

Genetically engineered mouse models for each specific TNBC subtype would greatly facilitate not only the analysis of subtype specific vulnerabilities but also provide immune-competent hosts to test potential ICB-based therapeutics. BL1 TNBC is modeled by targeted deletion of RB1 or PTEN together with p53 deletion or mutation^19,30–32^. Given the combined loss of all three tumor-suppressor genes in aggressive BL1 (this study), the generation of triple Rb/Pten/p53 triple mutant mouse model in the mammary gland is warranted. Notably, recent preclinical analysis in several laboratories point to the CHK1/WEE1/CDC25/Aurora Kinase pathway as a target for RB1/PTEN/TP53-deficient basal and mesenchymal TNBC subtypes^24,33^.

For BL2, a logical model would involve Akt1/PKBα overexpression on a p53 mutant background. Notably, expression of activated PKB/Akt1 in mammary epithelium provides a survival signal that cooperates with a mutant middle T antigen, defective in PI3K signaling, to promote tumor growth but does not promote metastatic progression^34^. Similarly, p53-R172H mutation increased mammary tumors in transgenic myristylated-AKT mice, but did not promote full tumor formation^35^. This activated AKT1 allele induces senescence, a barrier for tumorigenesis. Perhaps a BL2 TNBC model would require p53 mutation plus high wild-type AKT1/PKB expression either with other genes on the 14q32.3 amplicon (through chromosome engineering) or in combination with other oncogenic alterations that occur in BL2.

The tumor immune microenvironment is controlled by complex interaction with tumor cells and can be exploited therapeutically^36–38^. Indeed, one mechanism by which tumors evade the immune system involves suppression of cytotoxic T cells through expression of Programmed Death-Ligand 1 (PD-L1; cluster of differentiation 274, CD274). PD-L1 binds Programmed cell death protein 1 (PD-1, CD279) on the surface of cytotoxic CD8+ T cells, shutting down their ability to kill tumor cells. High expression of PD-L1 on tumor cells or in the TME, and expression of PD-1 receptor on immune cells, inversely correlates with survival of BC patients^39^. PD-L1 also inhibits CD4 T helper cells and promotes survival of regulatory anti-inflammatory, suppressive T regulatory (Treg) cells. As noted, a phase III trial on TNBC patients with PD-L1 positive tumors revealed a median OS of 27 months following anti-PD-L1 (Atezolizumab) plus nab-Paclitaxel treatment, compared with 15.5 months for nab-Paclitaxel only therapy^8^. While these are seminal results, the response was not durable and many patients, including those with high PD-L1 expression, showed only marginal response, and ultimately succumbed to their disease, albeit at a slower pace. This raises the question of whether specific subgroups of TNBC patients may respond better to anti-PD-L1 therapy.

A recent analysis of immune microenvironment of TNBC identified four groups on the basis of CD8+ T cells in the tumor core, stroma and epithelial compartments as well as expression of other immune determinants such as PD-L1, uncovering extensive complexity^40^. Here, we found that high expression of the PD-L1 receptor, PD1, alone identifies IM TNBC patients at high risk who succumb, on average, over 10 years sooner than patients with PD1-low tumors. The effect was not seen when all TNBC patients were considered, indicating that different immune factors impact survival in other TNBC subtypes. Our results suggest that IM patients with high PD1 expression may benefit from anti-PD1 or PD-L1 therapy. Interestingly, TGFβ was shown to attenuate tumour response to PD-L1 blockade by trapping cytotoxic T cells in the stroma, excluding them from the tumor parenchyma^41,42^. In this regard, our observation of high TGFβ signalling in BL2 TNBC points to these patients as excellent candidates for anti-PD-L1 plus anti-TGFβ combination therapy.

In conclusions, we report that TNBC subtypes, in particular BL1, BL2 and IM, can be further stratified on the basis of intrinsic oncogenic alterations. The effect of stratification is considerable with survival differences of more than 14, 2.75 and 10.5 years between high and low risk BL1, BL2 and IM patients, respectively. These poorest outcome subgroups within each subtype can now be identified and prioritized for aggressive, personalized therapy. The oncogenic alterations used to stratify these TNBC subtypes can be further used as targets for therapeutic interventions.

## Methods

### Datasets

Training set included 1302 BC/205 TNBCs from public mRNA dataset EGAD00010000434 and miRNA dataset EGAD00010000438 were obtained from EGAS00000000122^43^; CNA dataset was obtained from EGAS00001001753^44^ in which, 185 TNBCs have mutation data. In the validation set, a cohort containing 207 BC/44 TNBC from mRNA dataset GSE22219 and miRNA dataset GSE22216 were obtained from GSE22220^45^ and used to verify PTEN/miRNA subtypes. In addition, another validation cohort containing 146 TNBCs with mRNA and mutation data was obtained from METABRIC (https://www.cbioportal.org/study/summary?id=brca_metabric). mRNA data from the integrated 190 TNBC in the two validation cohorts were used to confirm TNBC subtypes expression in the training cohort.

### TNBC subtypes and pathway signatures

PTEN/miRNA groups^23^, TNBCType-6^3^, TNBCType-4^6^, PAM50 + Claudin^2^ and IntCluster^22^ subtypes were classified according to the methods in each original publication. Pathway activity of 25 oncogenic and tumor suppressor gene signatures^25,26^ and RB1-loss signature^24^ were calculated as described.

### Statistical analysis

Statistical analysis was performed using Prism Software (GraphPad Software, La Jolla, CA, USA). Differences in gene expression, pathway and signature activity, CNA, and mutation status between different groups were calculated using ANOVA and student t-test. For Kaplan-Meier survival analysis log-rank (Mantel-Cox) test was used to calculate P values and hazard ratios (HRs), and to compare survival curves. P value < 0.05 was considered significant and marked as *. P < 0.01, P < 0.001 and P < 0.0001 were marked as **, *** and ****, respectively. P > 0.05 was considered not significant (ns).

## Supplementary information

Supplemental Information

## Data Availability

All data generated and/or analyzed during this study are referenced or included in this published article.
