# Supplementary material for: Molecular stratification within triple-negative breast cancer subtypes
Source: Sci Rep. 2019 Dec 13;9:19107. doi: 10.1038/s41598-019-55710-w (PMC6911070; doi:10.1038/s41598-019-55710-w)
Supplement: Supplementary file 1 — Supplemental Information [file 41598_2019_55710_MOESM1_ESM.pdf]

SUPPLEMENTAL INFORMATION

Molecular stratification within triple-negative breast cancer subtypes

Dong-Yu Wang[1], Zhe Jiang[1], Yaacov Ben-David[2], James R. Woodgett[3] and Eldad Zacksenhaus[1, 4,*]

LEGENDS TO SUPPLEMENTAL FIGURES

Supplemental Figure 1. Impact of indicated pathway activity in specific TNBC subtypes on patient's survival. (A-B, D-E) Although significantly induced (Fig. 2), high pathway activity of E2F2, TGFβ, IFNγ, and EGFR did not impact clinical outcome in indicated subtypes. **(C)** High activity of IFNα pathway was significantly high in both training and validation sets (Figure 2F) and predicted poor clinical outcome in the training set.

Supplemental Figure 2. **Cancer gene mutations in TNBC subtypes**. Mutation spectrum of 135 genes that are often altered in breast cancer with at least one mutation in the 185 TNBC training set, classified by the 6 TNBC subtypes and ranked by mutated tumor number.

Supplemental Figure 3. **TP53 and PIK3CA mutations in TNBC subtypes**. **(A-B)** TP53 and PIK3CA mutations in the 185 TNBC training set (A) and 146 TNBC validation set (B). (**C-D**) PIK3CA mutation leads to poor prognosis in test and validation cohorts. (D) Combination of PIK3CA plus TP53 mutation has different effects on outcome in the two different cohorts.

Supplemental Figure 4. **Total CNAs in 205 TNBC subtypes**. Top, CNA spectrum of 22,544 CNA probes in 205 TNBC stratified by TNBC type. The bottom histogram shows the frequency of CNA gains and losses in each case and subtype.

Supplemental Figure 5. **Clustering of CNAs in TNBC subtypes**. ANOVA-selected 2,744 CNA probes and a cluster contained common gains (chr16p13.3, filled arrow on the right) in BL2 and a group of AKT-related gains (chr14q32.3, arrow on the right). Bottom, a group of CNAs including PTEN showing homozygous deletion in BL1 lesions.

Supplemental Figure 6. **Common gains on chr16p13.3 in BL2 subtype without outcome prediction**. (**A**) 12 CNAs on chr16p13.3 in BL2. **(B)** PDPK1 and miR3176 show significantly higher expression in BL2, but no impact on clinical outcome in TNBC or BL2. (**C**) No co-effect of PDPK1 or miR3176 in combination with PTEN-low mRNA, MYC CNA or PIK3CA mutation in BL2. (**D**) Six other microRNAs within the chr16p13.3 gain show no predictive power in BL2 TNBC.

Supplemental Figure 7. **Expression of known breast cancer genes with CNAs in TNBC subtypes**. (**A-B**) MYC and GATA3 show the most frequent copy number gain in BL1 and significantly predict clinical outcome in all TNBC subtypes but except BL1. **(C-D)** After AKT1, the most frequent CNA gain in BL2 includes AXIN1 and CREBBP. AXIN1 but not CREBBP copy number gain predicts a poor outcome in TNBC, but not in BL2. (**E-H**) Most frequent copy number loss of PTEN, ZFP36L1, PIK3R1 and FBXW7 were observed in BL1 but none showed a prediction power in TNBC or BL1.

Supplemental Figure 8. **Genes on the Chr14q32.3 amplicon and clinical outcome in BL2**. Next to AKT1 CNA (**Figure 4**), CDCA4 is most frequently gained in BL2 TNBC with a higher mRNA level and poor clinical prognosis. However, by mRNA expression CDCA4 expression was predictive in one of the two clinical cohorts. **(B)** Eight other genes that are gained within the chr14q32.3 amplicon predict poor clinical outcome in BL2.

Supplemental Figure 9. Expression of immune checkpoint genes, inflammation and MYC pathway in PTEN-low/miR-low (group "a") TNBC, ER$^+$, and HER2$^+$ subtypes. (**A**) mRNA expression of immune checkpoint and inflammation genes showing differences in group-a, TNBC, ER$^+$ and HER2$^+$ subtypes in 1296 BC in the training set. (**B-D**) MYC pathway activity and expression of CD47 and CD274 (PD-L1) is highest in group-a in training and validation sets. (**E**) Multiple

inflammation associated genes show highest mRNA expression in PTEN-low/miR-low (group “a”) TNBC.

Supplemental Figure 10. **A group of checkpoint and inflammation genes shw differential expression but insignificant predictive power. (A)** High CTLA4 expression in IM. (**B**) High CXCL8 expression in BL2. (**C**) High CCL2 expression in MSL.

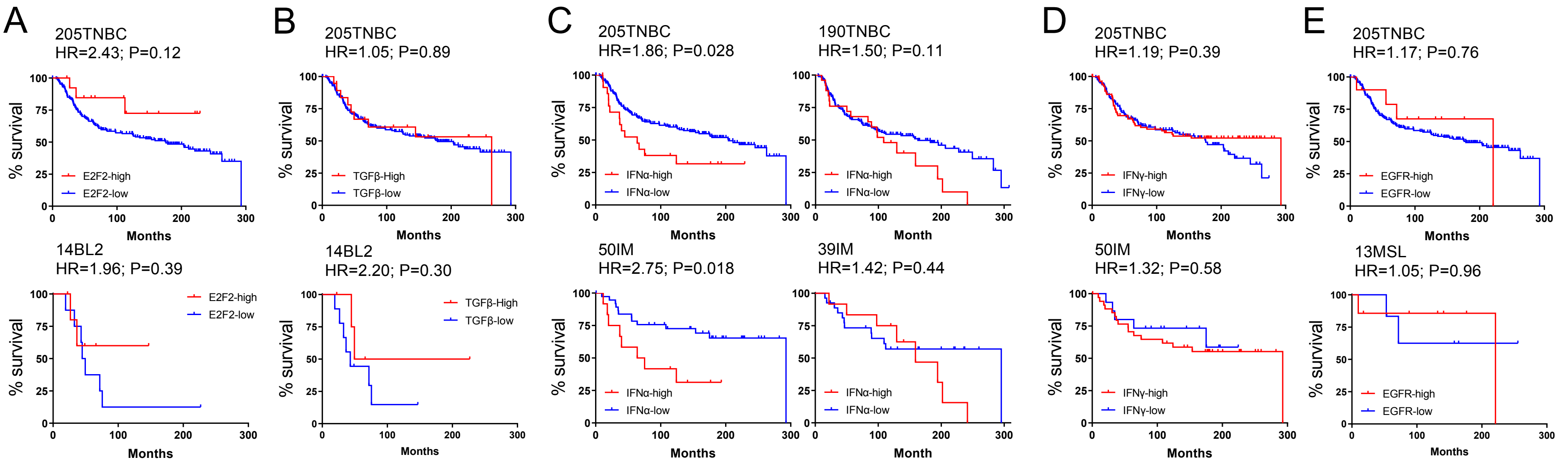

Supplemental Figure 1

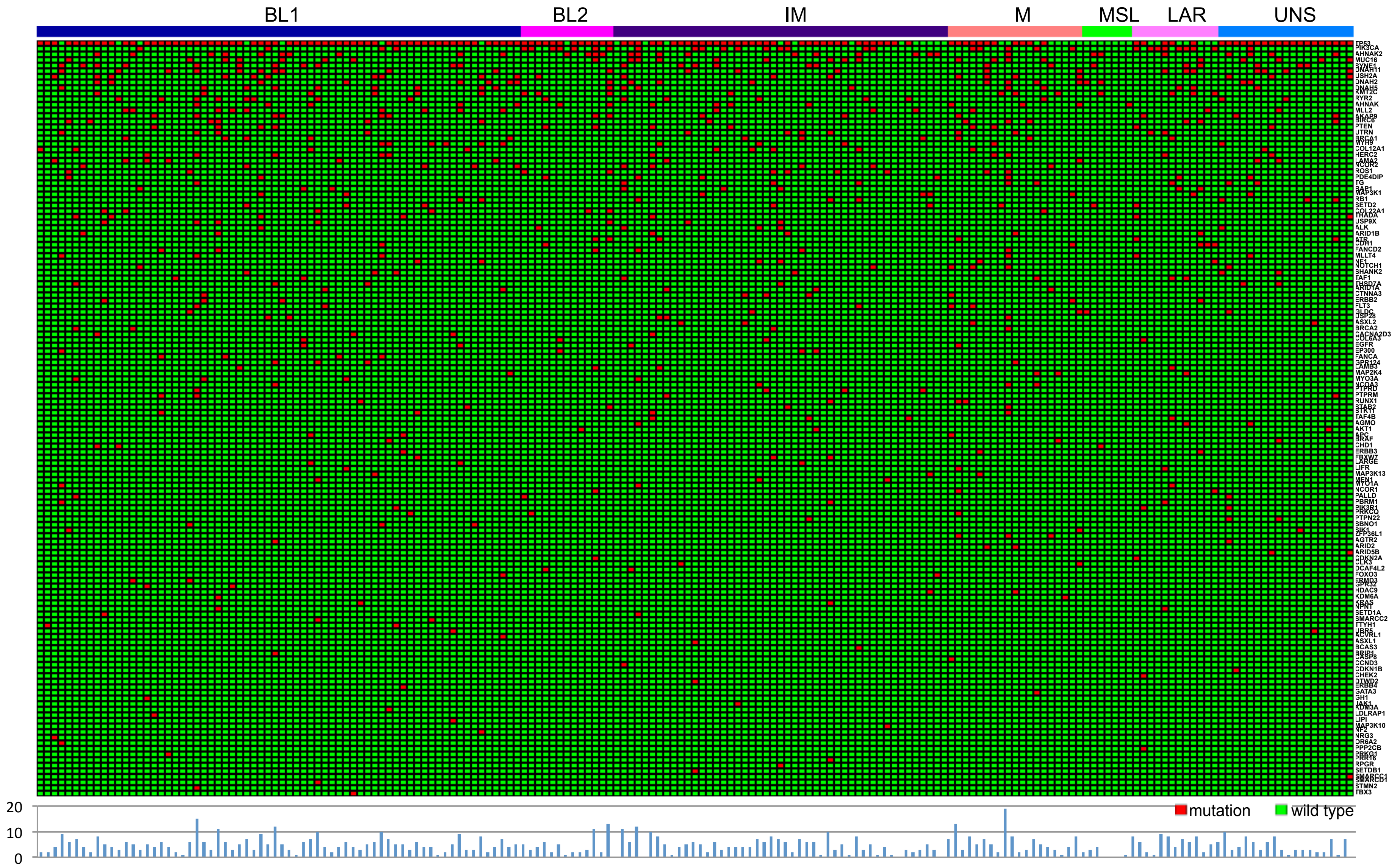

Supplemental Figure 2

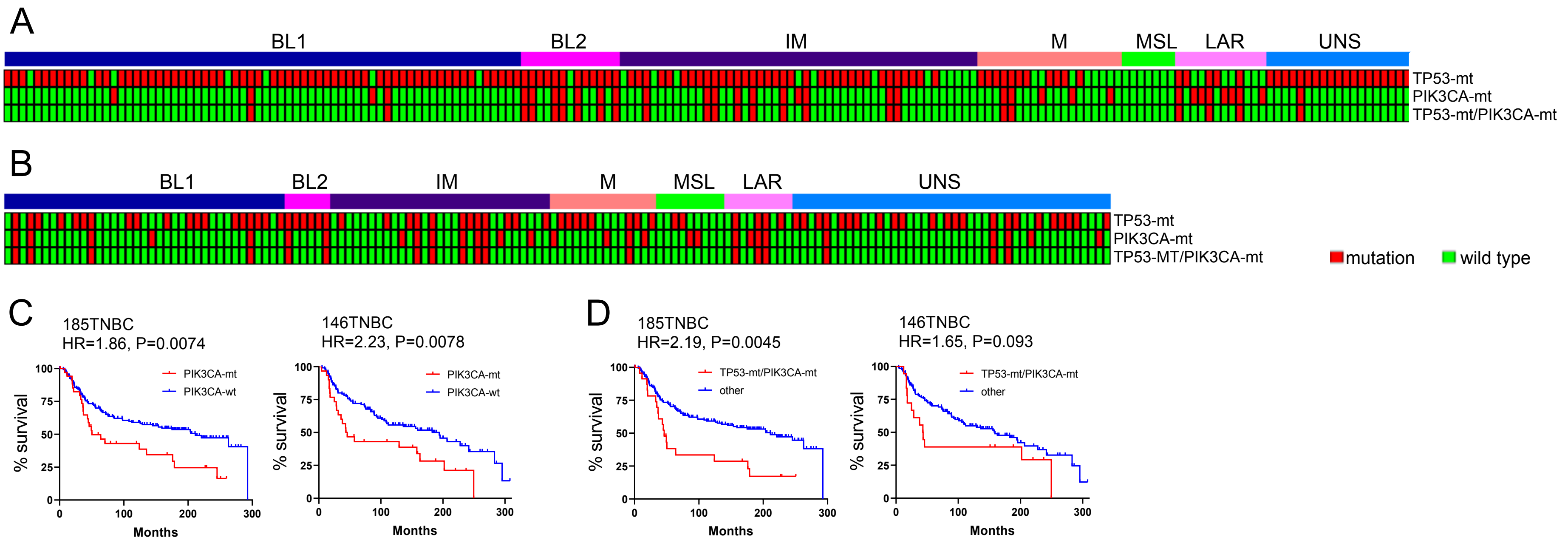

Supplemental Figure 3

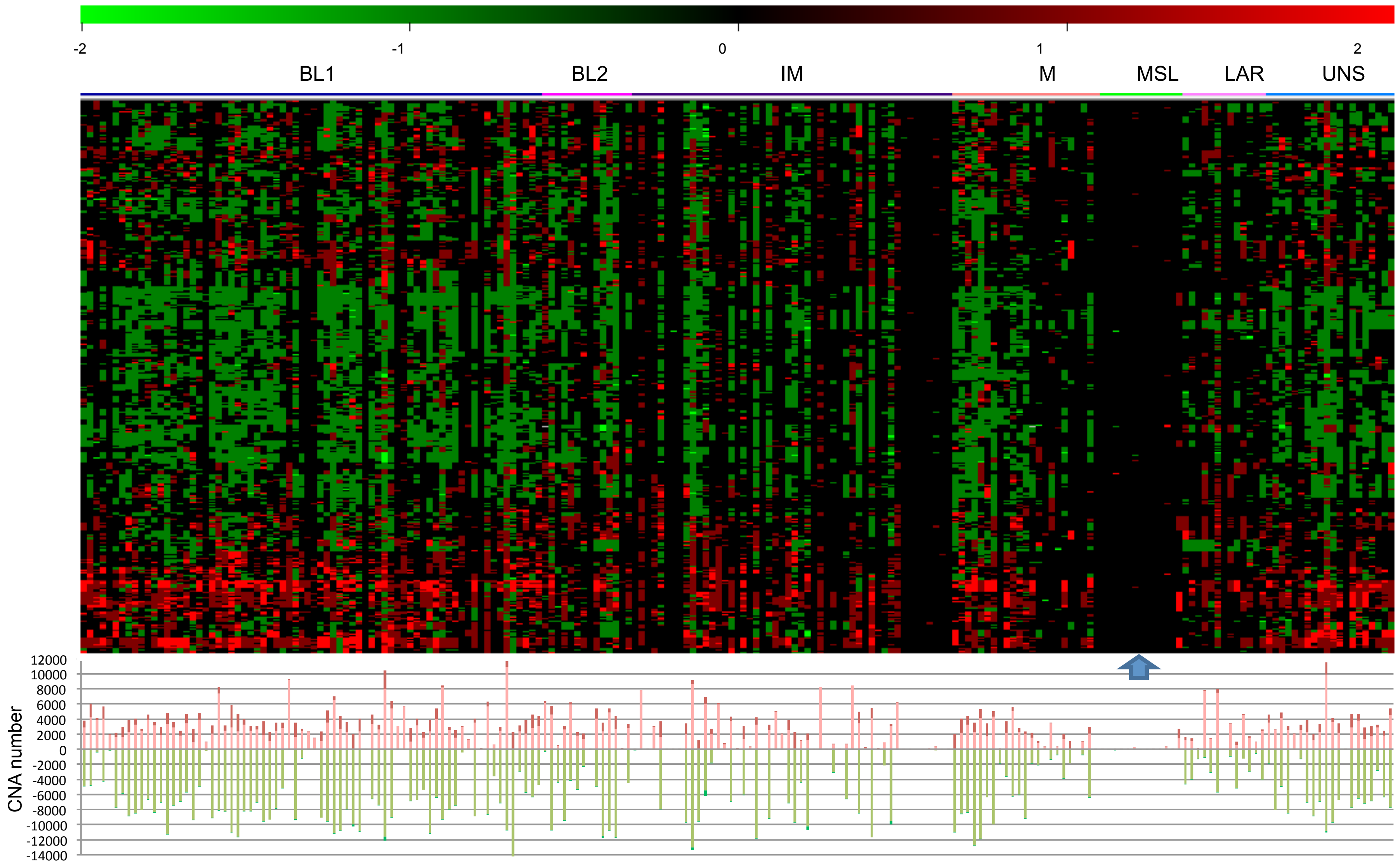

CNV: 2=high level amplification, 1=gain, 0=neutral, -1=hemizygous deletion, -2=homozygous deletion.

Supplemental Figure 4

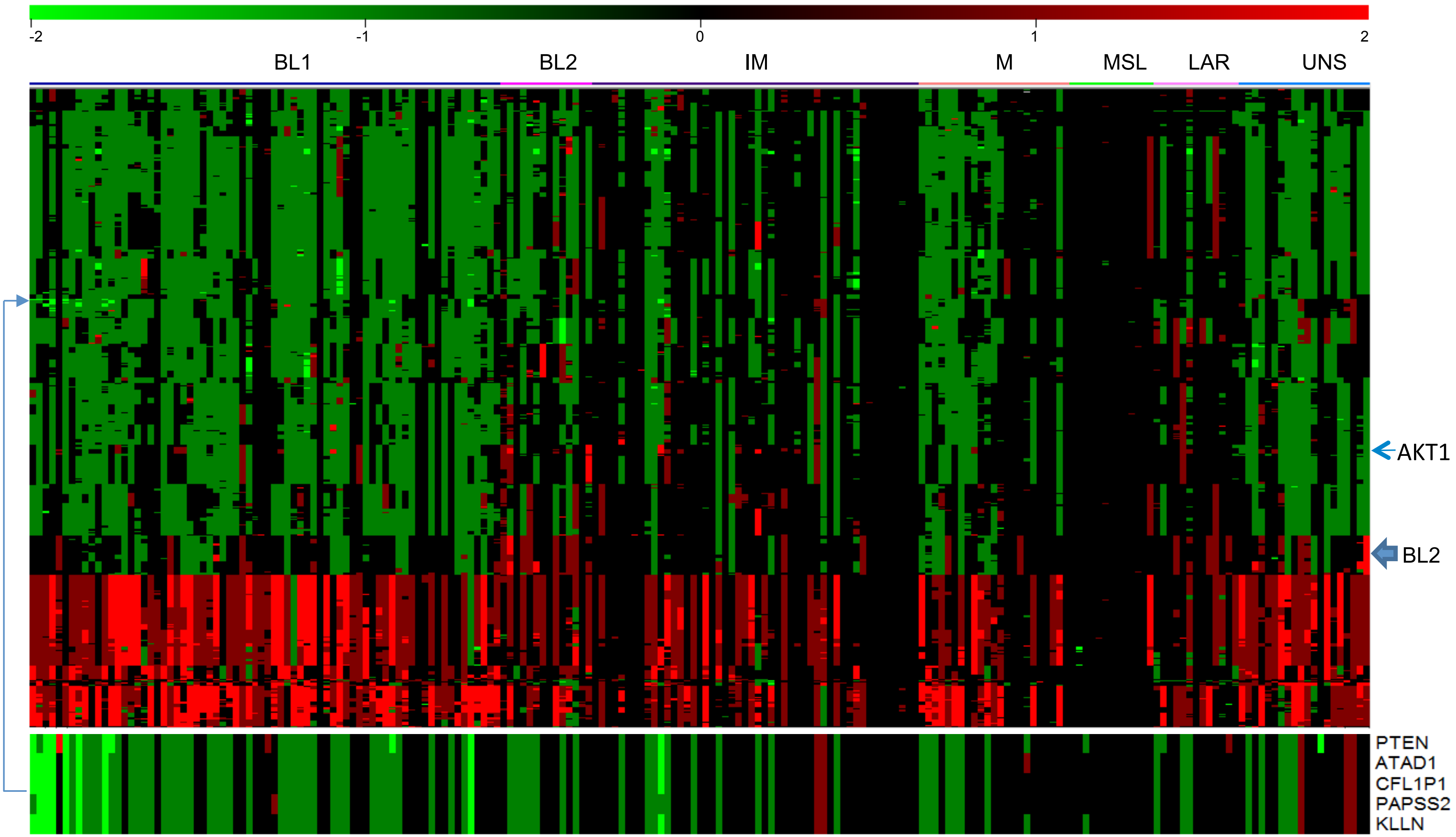

CNV: 2=high level amplification, 1=gain, 0=neutral, -1=hemizygous deletion, -2=homozygous deletion.

Supplemental Figure 5

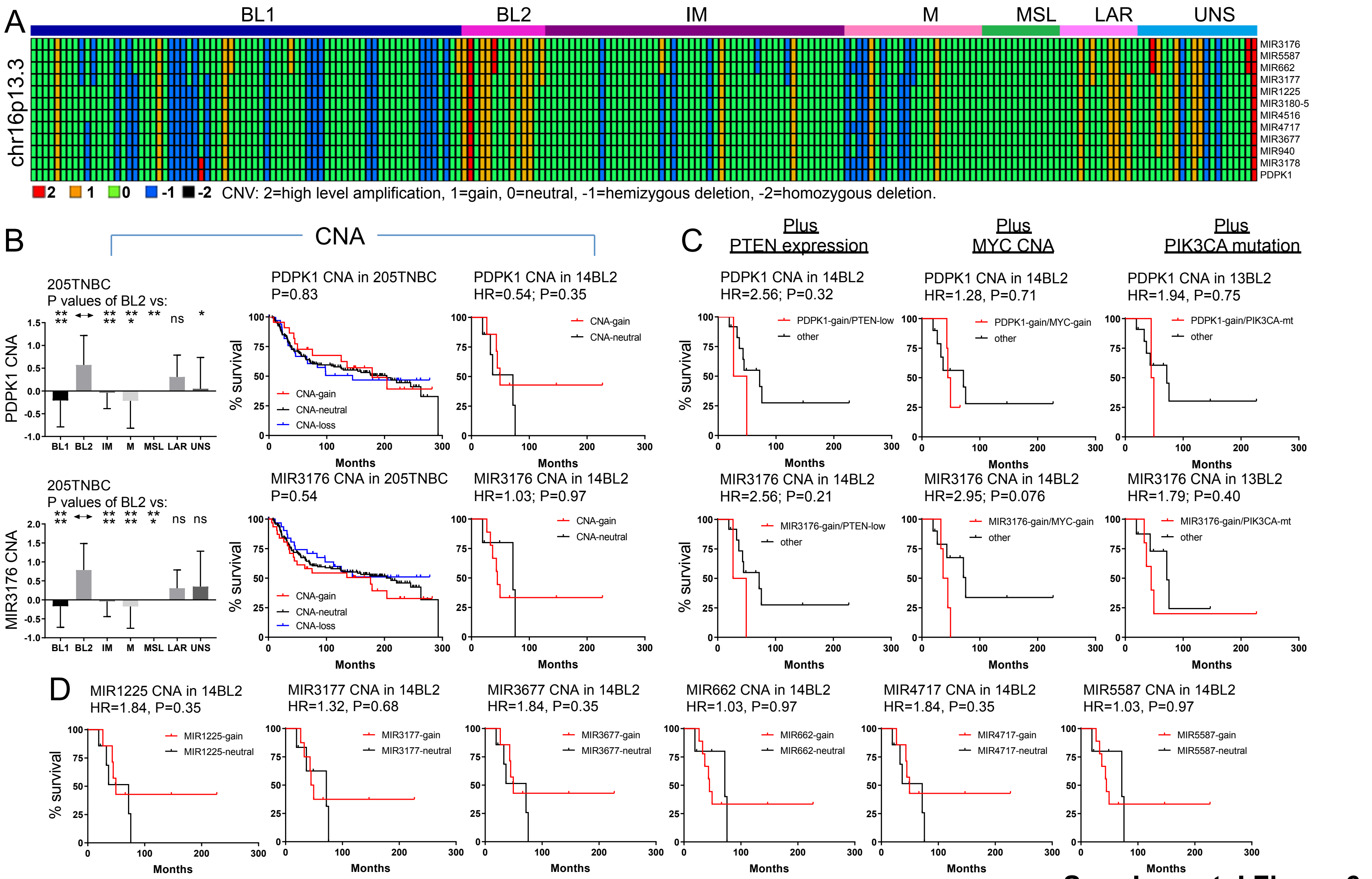

Supplemental Figure 6

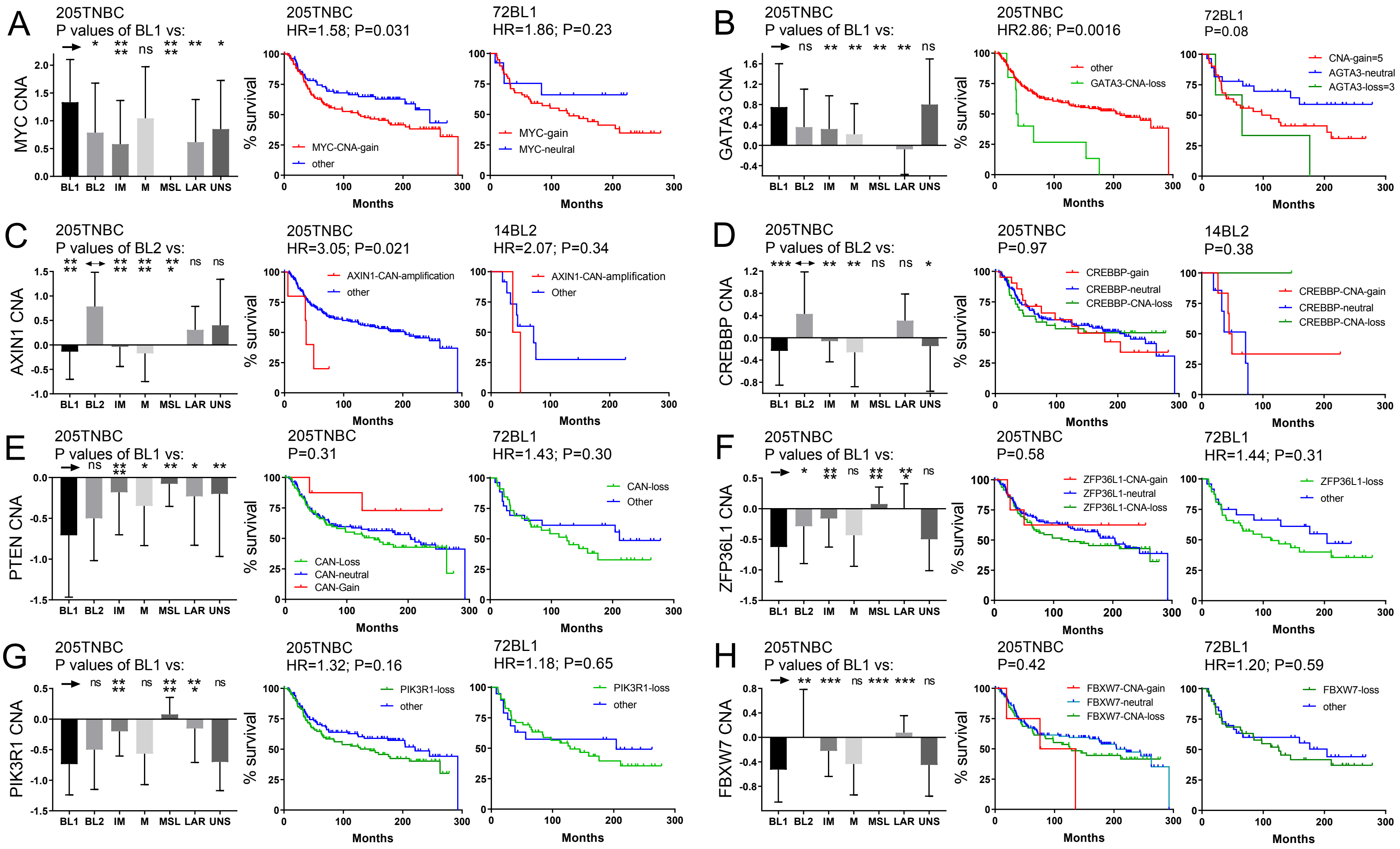

Supplemental Figure 7

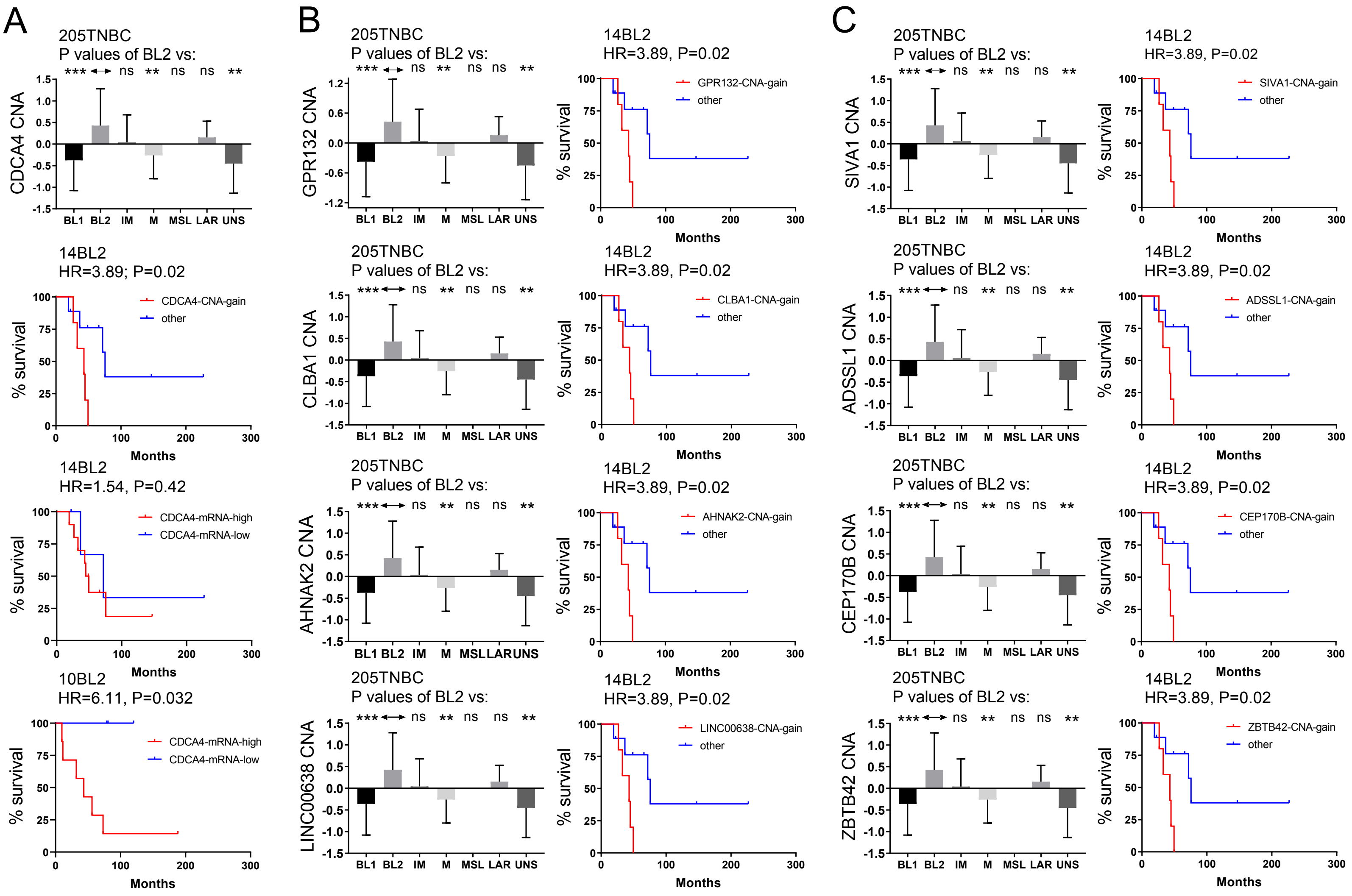

Supplemental Figure 8

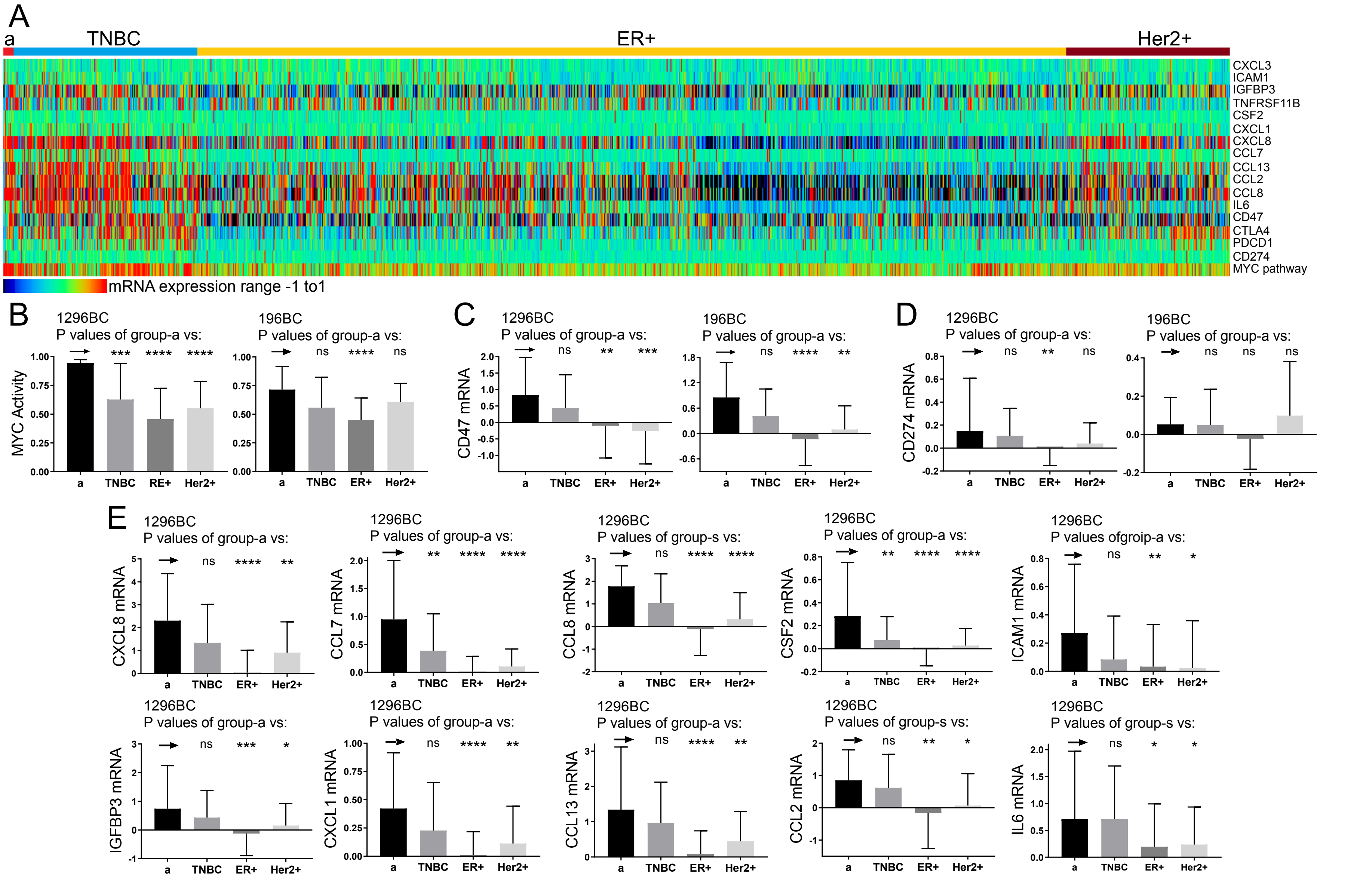

Supplemental Figure 9

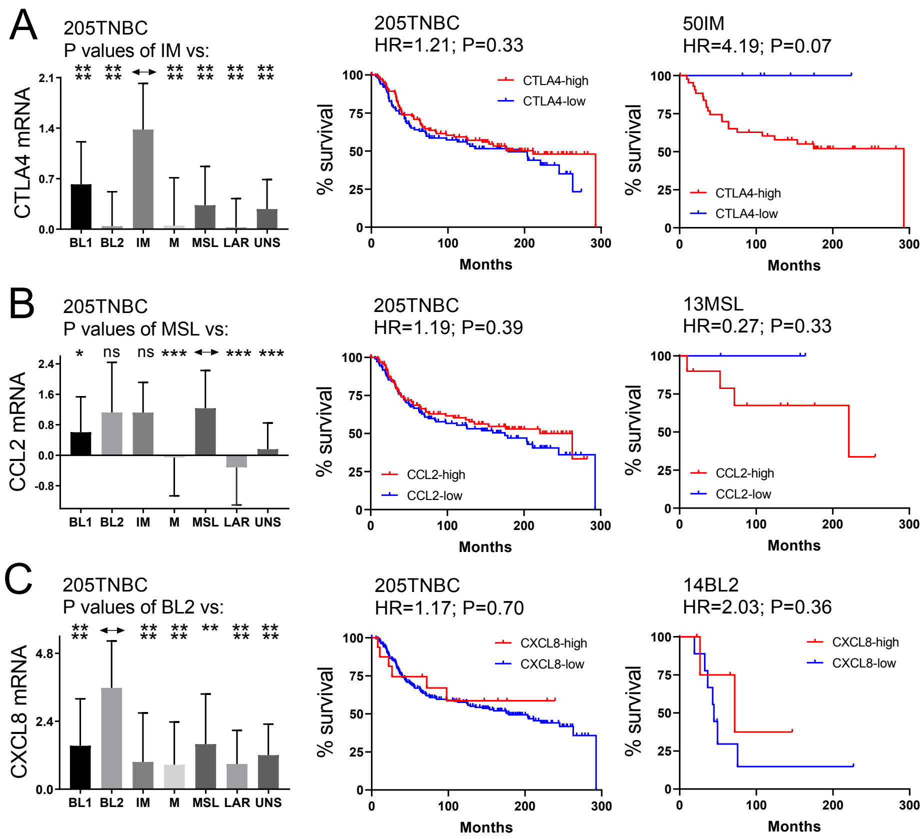

Supplemental Figure 10
